# Comparative neuroprotective and cognitive effects of human umbilical cord mesenchymal stem cells, secretome, and exosomes in a rat model of pilocarpine-induced epilepsy

**DOI:** 10.14202/vetworld.2026.2606-2618

**Published:** 2026-06-25

**Authors:** Sri Hastuti, Indra Bactiar, Yetty Ramli, Imran Imran, Rinaldi Idroes

**Affiliations:** 1 Doctoral Program, Faculty of Medicine, Universitas Syiah Kuala, Banda Aceh, 23111, Indonesia; 2 Department of Neurology, General Hospital dr. Zainoel Abidin, Banda Aceh, 23111, Indonesia; 3 PT Tristem Medika Indonesia, Cell Therapy Centre, Solo, Indonesia; 4 Department of Neurology, Faculty of Medicine, Universitas Indonesia, Jakarta, 10430, Indonesia; 5 School of Mathematics and Applied Sciences, Universitas Syiah Kuala, Banda Aceh 23111, Indonesia; 6 Department of Pharmacy, Faculty of Mathematics and Natural Sciences, Universitas Syiah Kuala, Banda Aceh, 23111, Indonesia

**Keywords:** cognitive function, epilepsy, exosomes, human umbilical cord mesenchymal stem cells, neuroprotection, secretome, spatial learning, stem cell therapy

## Abstract

**Background and Aim:**

Epilepsy is a chronic neurological disorder characterized by recurrent seizures, neuronal degeneration, and cognitive impairment. Current antiepileptic therapies mainly control seizures and do not effectively prevent epileptogenesis or restore neurological function. Human umbilical cord mesenchymal stem cells (HuMSCs) and their cell-free derivatives, including secretome and exosomes, have emerged as promising regenerative therapies. This study aimed to compare the neuroprotective and cognitive effects of HuMSCs, secretome, and exosomes in a rat model of pilocarpine-induced epilepsy.

**Materials and Methods:**

Thirty male Wistar rats were randomly allocated into five groups (n = 6/group): healthy control, untreated epilepsy, HuMSC-treated epilepsy, secretome-treated epilepsy, and exosome-treated epilepsy. Epilepsy was induced by intraperitoneal pilocarpine administration following scopolamine pre-treatment. Treatments were administered intravenously on days 14, 28, and 42 after induction. Cognitive performance was assessed using the Morris Water Maze test between days 50 and 56. Histopathological examination of the hippocampus, immunohistochemical evaluation, and quantitative polymerase chain reaction analysis of synaptic vesicle glycoprotein 2A (SV2A) and SRY-box transcription factor 10 (SOX10) expression were performed to determine therapeutic efficacy.

**Results:**

HuMSC therapy significantly reduced hippocampal neuronal damage compared with untreated epilepsy, secretome, and exosome groups (p < 0.05). The mean number of damaged neurons was lowest in the HuMSC-treated group and approached normal control values. HuMSC treatment restored SV2A and SOX10 expression to levels comparable with those of healthy controls, indicating improved synaptic integrity and glial support. In contrast, secretome treatment produced moderate improvement, whereas exosome treatment showed limited therapeutic benefit. Cognitive assessment revealed significantly shorter escape latency and superior spatial learning performance in the HuMSC-treated rats compared with all other epilepsy groups (p < 0.05). Behavioral improvements were consistent with the molecular and histopathological findings, demonstrating enhanced neuroprotection and functional recovery following HuMSC administration.

**Conclusion:**

HuMSC therapy provided superior neuroprotective, molecular, and cognitive benefits compared with secretome and exosome treatments in a pilocarpine-induced epilepsy model. These findings support the potential of HuMSCs as a regenerative therapeutic strategy for epilepsy and suggest that intact stem cells may offer greater therapeutic efficacy than their cell-free derivatives. Further studies are required to optimize treatment protocols and evaluate long-term translational potential.

## INTRODUCTION

Epilepsy is a chronic neurological disorder characterized by excessive and abnormal electrical activity in the brain, which can lead to progressive neuronal damage [1]. Its incidence varies globally, ranging from 33.3 to 82 cases per 100,000 people annually in Western countries and up to 187 cases per 100,000 people in developing nations. In children, the incidence is approximately 40 cases per 100,000 population each year [2, 3]. Current treatments for epilepsy primarily focus on symptom management rather than addressing the underlying causes of the disease or preventing its progression.

Stem cell therapy has emerged as a promising approach for treating neurological disorders, including epilepsy [4]. Epileptogenesis, the process by which a normal brain develops epilepsy after events such as prolonged seizures, stroke, or brain injury, involves multiple mechanisms, including neuroinflammation, disruption of the blood–brain barrier, neuronal loss, and increased neuronal excitability [5–7]. Additional factors, such as abnormal neurogenesis, altered synaptic plasticity, and neural circuit remodeling, particularly in the hippocampus, further contribute to the development of chronic seizures [8]. The complexity of these processes highlights the need for therapeutic strategies that not only suppress seizures but also target the underlying pathological mechanisms responsible for epileptogenesis.

Among various stem cell options, human umbilical cord mesenchymal stem cells (HuMSCs) are particularly attractive for their multipotency and capacity to differentiate into multiple cell types [9–12]. HuMSCs, especially those derived from Wharton’s jelly, offer several advantages, including noninvasive harvesting, high proliferative capacity, low immunogenicity, and minimal ethical concerns [13]. Previous studies have demonstrated their neuroprotective and anti-inflammatory effects, as well as their ability to improve motor and cognitive outcomes in preclinical models of ischemia and epilepsy [14].

Stem cell therapies exert their therapeutic effects through two principal mechanisms: direct cell replacement through differentiation and the release of bioactive molecules collectively referred to as the secretome, which supports tissue repair and modulates inflammatory responses [15, 16]. The secretome comprises a diverse array of soluble proteins, growth factors, cytokines, and extracellular matrix components that play essential roles in cell signaling, regeneration, and immune regulation [17, 18]. Despite its therapeutic potential, direct comparisons between secretome-based therapy and conventional stem cell transplantation remain limited [19–23].

A key component of the secretome is exosomes, which are small extracellular vesicles capable of crossing the blood–brain barrier and mediating intercellular communication [24, 25]. Exosomes derived from HuMSCs possess neuroprotective, regenerative, and anti-inflammatory properties, making them promising candidates as both biomarkers and therapeutic agents for epilepsy [26]. Nevertheless, the application of HuMSC-derived secretome and exosome therapy in epilepsy remains underexplored, particularly in countries such as Indonesia, where the burden of epilepsy remains substantial [8].

Conventional epilepsy treatment continues to rely heavily on antiepileptic drugs, which primarily alleviate symptoms without preventing epileptogenesis. These drugs may also cause adverse effects, particularly in pediatric patients, underscoring the need for safer, more effective therapeutic approaches that promote neural repair and regeneration [16]. Cell-based therapies, including HuMSCs and their cell-free derivatives, represent ethically acceptable and minimally invasive alternatives; however, their clinical and experimental utilization remains limited despite growing evidence supporting their therapeutic potential [11].

Although HuMSCs, secretome, and exosomes have individually demonstrated neuroprotective and regenerative properties in various neurological disorders, substantial knowledge gaps remain regarding their relative therapeutic efficacy in epilepsy. Most previous studies have evaluated these interventions independently, using different experimental conditions, outcome measures, and disease models, thereby limiting direct comparison of their biological and therapeutic effects. Consequently, it remains unclear whether intact HuMSCs provide superior neuroprotection, synaptic preservation, and cognitive recovery compared with their cell-free derivatives. Furthermore, limited evidence is available on the comparative effects of these therapies on molecular markers of synaptic integrity and glial support in epilepsy.

Therefore, the present study was designed to address this research gap by directly comparing the neuroprotective and cognitive effects of HuMSCs, secretome, and exosomes in a rat model of pilocarpine-induced epilepsy using integrated histopathological, molecular, and behavioral assessments. We hypothesized that HuMSC therapy would provide greater neuroprotective and cognitive benefits than secretome and exosome treatments because of its combined regenerative and paracrine mechanisms. To the best of our knowledge, this study is among the few investigations to directly compare HuMSCs, secretome, and exosomes within the same epilepsy model using complementary molecular, histological, and behavioral endpoints. The findings are expected to provide valuable insight into the relative efficacy of cell-based and cell-free regenerative therapies and to support the development of more effective therapeutic strategies for epilepsy.

## MATERIALS AND METHODS

### Ethical approval

The study protocol was reviewed and approved by the Research Ethics Commission, Faculty of Veterinary Medicine, Universitas Syiah Kuala, Banda Aceh, Indonesia (Approval No. 172/KEPH/X/2022; approved on October 24, 2022). All experimental procedures involving animals were conducted in accordance with the institutional guidelines for the care and use of laboratory animals and complied with internationally accepted principles for animal welfare and ethical research. Thirty male Wistar rats (*Rattus norvegicus*) were housed under standardized environmental conditions with ad libitum access to feed and water throughout the study. Every effort was made to minimize animal suffering, stress, and discomfort during epilepsy induction, treatment administration, behavioral testing, sample collection, and euthanasia procedures. Animals were monitored daily for health status and signs of distress, and humane endpoints were applied when necessary. Euthanasia was performed at the designated study endpoints using approved humane procedures before tissue collection for histopathological and molecular analyses.

### Study period and location

The study was conducted from May to November 2023 at the Faculty of Veterinary Medicine and affiliated research facilities of Universitas Syiah Kuala, Banda Aceh, Indonesia. Animal experiments, behavioral assessments, histopathological examinations, immunohistochemical analyses, and molecular evaluations were performed in accordance with standardized laboratory protocols. Preparation of HuMSCs, secretome, and exosomes was performed at the Tristem Medika Indonesia Laboratory in Solo, Indonesia.

### Study design

This study integrated behavioral, histopathological, and molecular analyses within a single experimental framework to provide a comprehensive evaluation of therapeutic effects. A randomized controlled experimental design with post-intervention outcome assessment was employed.

Thirty male Wistar rats (*Rattus norvegicus*), aged 6 weeks and weighing 104–165 g, were housed under a 12-h light/dark cycle at 24°C with ad libitum access to food and water. Male rats were selected to reduce biological variability associated with sex hormones and to provide a more uniform baseline for comparative assessment among treatment groups. Inclusion criteria consisted of healthy age-matched male rats. Exclusion criteria included pre-existing illnesses or abnormal conditions prior to the experiment. Dropout criteria included death during the study period, severe illness, or failure to meet the inclusion criteria.

The animals were randomly allocated into five groups: (1) healthy control group (normal rats without pilocarpine induction or treatment), (2) epilepsy group (pilocarpine-induced rats without treatment), (3) HuMSC-treated epilepsy group, (4) secretome-treated epilepsy group, and (5) exosome-treated epilepsy group. The sample size (n = 6 per group) was determined based on feasibility considerations and standard practice in exploratory animal studies involving multiple treatment groups, enabling statistical comparison of therapeutic outcomes. All groups were maintained under identical housing, induction, treatment, and assessment conditions throughout the study.

Epilepsy was induced with pilocarpine (160 mg/kg body weight [BW]) on day 0. Treatments (HuMSCs, secretome, or exosomes) were administered on days 14, 28, and 42 after induction. Cognitive function was assessed using the Morris Water Maze test between days 50 and 56. On day 56, animals were euthanized for organ collection, histopathological examination, and molecular analysis.

The selected intervention and assessment time points were based on reported pathological changes following pilocarpine-induced status epilepticus and previous studies demonstrating the therapeutic potential of HuMSC transplantation in epilepsy models. Interventions were initiated after epilepsy induction and administered repeatedly to evaluate therapeutic effects during disease progression, whereas behavioral and molecular assessments were performed at later stages to capture long-term therapeutic outcomes.

Previous studies have shown that HuMSC transplantation attenuates edema in the ventricles, hippocampus, and piriform cortex at days 8 and 29 following status epilepticus. Therefore, treatments were initiated after the acute and peak edema phases (day 14) and repeated during the subacute and chronic phases to evaluate sustained therapeutic effects. Behavioral and molecular assessments were conducted after day 50 to assess outcomes beyond edema resolution.

### Induction of epilepsy

Epilepsy was induced through a single intraperitoneal injection of pilocarpine (160 mg/kg BW) according to previously optimized protocols [27]. Control rats received sodium chloride solution (NaCl) instead of pilocarpine. Seizure activity was monitored using the Racine scale [28], and only animals reaching stages 3–5 were included to ensure successful and consistent induction of epilepsy across groups.

### Treatment administration

HuMSCs were administered at a dose of 2 × 10^5^ cells, suspended in 0.5 mL of phosphate-buffered saline (PBS), via intravenous injection into the coccygeal vein. Secretome was administered at a dose of 100 μg/kg BW in 0.5 mL PBS through tail-vein injection, whereas exosomes were administered at a dose of 100 μg/kg total protein in 0.5 mL PBS through tail-vein injection. These doses were selected in accordance with standardized laboratory protocols established for this experimental model and were applied consistently across all treated groups.

All therapeutic preparations were suspended in PBS and administered using the same injection volume and route to ensure consistency among treatment groups. The intravenous route was selected to facilitate systemic distribution and repeated administration of all therapeutic preparations using a minimally invasive and standardized approach. This route also enabled direct comparison between cell-based and cell-free therapies under equivalent delivery conditions.

### HuMSC, exosome, and secretome preparation

HuMSCs, secretome, and exosomes were prepared by Tristem Medika Indonesia Laboratory (Solo, Indonesia). HuMSCs were isolated from Wharton’s jelly using a combination of mechanical and enzymatic dissociation, followed by explant culture. Cells were cultured in α-minimum essential medium (α-MEM; Thermo Fisher Scientific, Waltham, MA, USA) supplemented with 10%–20% fetal bovine serum (FBS; Thermo Fisher Scientific) and harvested at approximately 80% confluence, as previously described [28].

During *in vitro* expansion, cultured cells exhibited plastic adherence and fibroblast-like morphology, consistent with MSC characteristics. Secretome and exosomes were obtained from cultured HuMSCs and prepared in accordance with standardized laboratory procedures for therapeutic administration. However, detailed phenotypic validation of HuMSCs and molecular characterization of the secretome and exosomes, including specific markers, particle-size distribution, and morphology, were not evaluated in the present study.

### Biomolecular analysis (quantitative polymerase chain reaction [qPCR])

For biomolecular analysis, hippocampal tissue was collected immediately after euthanasia and separated from the brain under sterile conditions. The hippocampus was homogenized and processed for total RNA extraction according to standard molecular biology procedures. The isolated RNA was subsequently used for complementary DNA synthesis, followed by qPCR analysis.

qPCR was performed under the following cycling conditions: initial denaturation at 95°C for 10 min, followed by 40 cycles of 95°C for 15 s, 60°C for 30 s, and 72°C for 30 s [29]. Relative expression of SRY-box transcription factor 10 (SOX10) and synaptic vesicle glycoprotein 2A (SV2A) was assessed using Glyceraldehyde-3-phosphate dehydrogenase (GAPDH) as the internal reference gene. Gene expression analysis was conducted under identical amplification conditions for all samples to enable comparison among groups.

### GAPDH

Forward: 5′-TGCACCACCAACTGCTTAGC-3′

Reverse: 5′-GGCATGGACTGTGGTCATGA-3′

### SOX10

Forward: 5′-CCACCTGCAGGATGAGAACT-3′

Reverse: 5′-TGGGACCTGATGTAGGGTTG-3′

### SV2A

Forward: 5′-ACGGAAGTGGGACAGTTGAC-3′

Reverse: 5′-TGGTGTTGGTAGGCAGTGAA-3′

SV2A was selected as a marker of synaptic function because of its established role in neurotransmitter release and its relevance as a target of antiepileptic drugs. SOX10 was selected as a marker of glial cell development and myelination due to its role in neural support and repair. Together, these biomarkers provide insight into both neuronal and glial components of epileptic pathology and therapeutic response.

### Immunohistochemical analysis

Hippocampal sections (Cornu ammonis 1 region) were fixed in 10% buffered neutral formalin and stained using the Hematoxylin and Eosin Stain Kit (Vector Laboratories, Newark, CA, USA; H-3502). Primary antibodies included SOX10 rabbit polyclonal antibody (Abcam, Cambridge, UK; ab155279; dilution 1:200) and SV2A mouse monoclonal antibody (Cell Signaling Technology, Danvers, MA, USA; 13111S; dilution 1:250). Staining intensity was evaluated using an ordinal scoring system (0 = no staining, 1 = weak staining, 2 = moderate staining, and 3 = strong staining) across five microscopic fields per sample [30, 31].

### Histopathological examination

Histological examination focused on the hippocampus to assess structural alterations following seizure induction. One rat from each group was euthanized on days 14, 28, and 42 for hippocampal tissue collection, whereas the remaining animals were euthanized on day 56 at the end of the observation period.

The hippocampus was fixed in 10% buffered neutral formalin and processed for histopathological analysis. Brain sections were stained with hematoxylin and eosin and examined using a Meiji Techno trinocular microscope (Meiji Techno Co., Ltd., Saitama, Japan) equipped with Top View software at 100× magnification. Detailed measurements of myelin thickness were performed at 400× magnification across five fields of view.

Histopathological evaluation was performed using predefined morphological criteria applied consistently across all samples. Neuronal damage was estimated by identifying cells with pyknotic nuclei, shrunken cytoplasm, and disrupted architecture in five non-overlapping fields per section. The number of damaged cells per field was recorded and averaged to estimate the extent of tissue damage. Histological parameters assessed included axonal myelination, synaptogenesis, neurogenesis, sclerosis, myelin sheath thickness, and neuronal death.

### Morris Water Maze test

The Morris Water Maze test was used to evaluate spatial learning and memory associated with hippocampal function in rats with pilocarpine-induced epilepsy. Following completion of all interventions, rats were euthanized 10 days after behavioral testing for organ collection, with emphasis on hippocampal evaluation.

The test was conducted in a circular pool measuring 150 cm in diameter and 50 cm in depth with a black bottom and filled with musky-green water maintained at 25 ± 2°C. A 10 × 10 cm polycarbonate platform was positioned 1 cm below the water surface in a fixed location. Rats underwent four training sessions per day and were allowed to swim freely for 60 s from different quadrants of the pool. Animals unable to locate the platform within the allotted time were guided to it and allowed to remain there for 20 s. A 15-min interval was maintained between trials. Sessions were recorded, and the primary outcome measures were escape latency and distance traveled, which served as indicators of spatial learning and memory. The same testing procedure was applied consistently across all experimental groups [32].

### Statistical analysis

All data are expressed as mean ± SD. Normality was assessed using the Shapiro–Wilk test, whereas homogeneity of variance was evaluated using Levene’s test. One-way analysis of variance followed by Duncan’s post hoc test was used to determine differences among groups. Statistical significance was defined as p < 0.05.

## RESULTS

### Effects on the immunohistochemical profile

**Figure 1:**
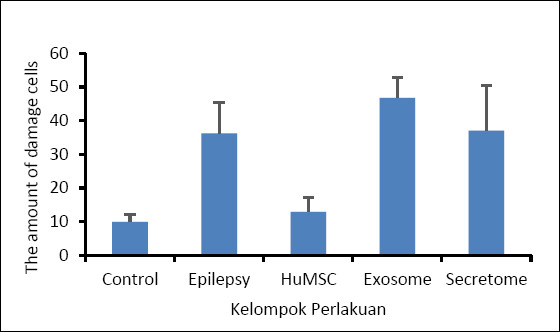
Histogram showing the mean number of damaged brain cells in epilepsy-model rats across five groups: control, epilepsy, HuMSC, secretome, and exosome. Results are based on Duncan’s post hoc test. Different superscript letters (a, b, c) indicate statistically significant differences among groups (p < 0.05). Statistical significance was assessed using one-way ANOVA followed by Duncan’s post hoc test. Differences were compared across all groups, with significance determined relative to both the epilepsy group (disease model) and the healthy control group.

The study results indicated that the highest mean number of damaged cells in the brain tissue of epilepsy-model rats was observed in the exosome-treated group (46.80 ± 6.08), followed by the secretome group (37.00 ± 13.52), epilepsy group (36.20 ± 9.22), HuMSC-treated group (12.93 ± 4.33), and control group (9.93 ± 2.21). Normality was assessed using the Shapiro–Wilk test, whereas homogeneity was evaluated using Levene’s test. Both tests showed p > 0.05, confirming that the data were normally distributed and homogeneous. Therefore, one-way analysis of variance (ANOVA) was performed. Duncan’s post hoc test revealed significant differences among groups (p < 0.05), as shown in Figure 1, where different superscript letters indicate statistically significant differences.

Interestingly, the exosome-treated group exhibited a higher number of damaged neurons than the untreated epilepsy group. This finding should be interpreted with caution and may be influenced by factors such as dose, biodistribution, or delivery efficiency after intravenous administration. Because these parameters were not directly evaluated in the present study, further investigations are required to clarify the underlying mechanisms. Although HuMSC-derived exosomes contain neuroprotective molecules such as miR-124 and various growth factors, their therapeutic efficacy depends substantially on concentration, biodistribution, and the ability to cross the blood–brain barrier. Furthermore, unlike intact HuMSCs, exosomes cannot differentiate into neural or glial cells and therefore lack direct regenerative and antiapoptotic capabilities. Consequently, the observed findings may be associated with differences in exosome delivery efficiency or biological activity, although these mechanisms remain to be elucidated.

**Figure 2:**
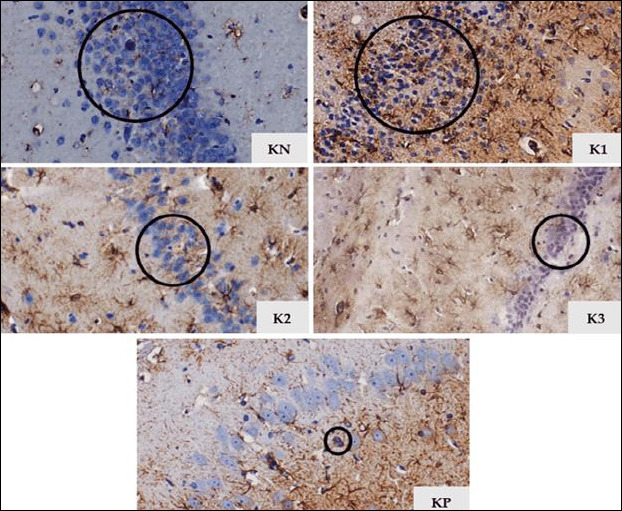
Immunohistochemical staining of GFAP protein expression in hippocampal brain tissue, counterstained with hematoxylin and eosin and observed under 400× magnification. Groups: (KN) Control, (K1) MSC-treated, (K2) secretome-treated, (K3) exosome-treated, and (KP) pilocarpine-induced. Circled areas highlight myelin presence. Background staining intensity was assessed using an intensity-scoring system; darker brown staining indicates stronger antibody binding and suggests increased GFAP expression.

Additionally, Figure 2 shows immunohistochemical staining for Glial Fibrillary Acidic Protein (GFAP) in hippocampal tissue, counterstained with hematoxylin and eosin. Brown staining intensity was notably higher in the exosome and secretome groups, indicating elevated GFAP expression compared with the control and HuMSC groups.

### Effects on SV2A expression

**Figure 3:**
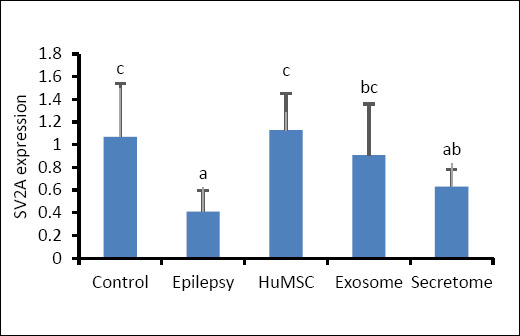
Histogram of Duncan’s post hoc test results showing the mean expression of SV2A in the brain tissue of epilepsy-model rats treated with control, epilepsy, HuMSC, secretome, and exosome therapies. The superscripts a, b, and c above the histogram indicate significant differences (p < 0.05). Statistical significance was calculated using one-way ANOVA followed by Duncan’s post hoc test, with comparisons performed across all groups.

SV2A expression was significantly decreased in the epilepsy group (0.41 ± 0.19) compared with the control group (1.07 ± 0.46). Expression was partially restored following treatment with HuMSCs (1.12 ± 0.32), secretome (0.91 ± 0.45), and exosomes (0.63 ± 0.15). The HuMSC-treated group exhibited expression levels comparable to those of the control group. These differences were statistically significant (p < 0.05; Figure 3), indicating that HuMSC therapy restored synaptic protein expression.

### Effects on SOX10 expression

**Figure 4:**
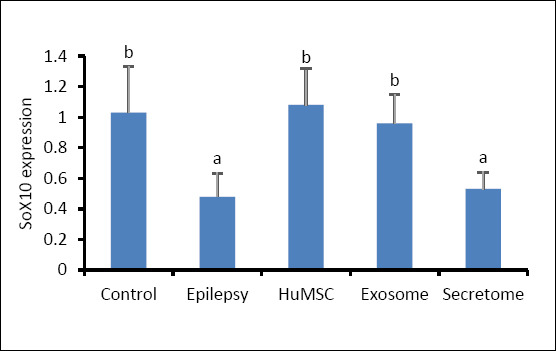
Histogram of Duncan’s post hoc test results showing the mean expression of SOX10 in the brain tissue of epilepsy-model rats treated with control, epilepsy, HuMSC, secretome, and exosome therapies. The superscripts a, b, and c above the histogram indicate significant differences (p < 0.05).

Figure 4 shows the expression levels of SOX10 across the experimental groups. The lowest SOX10 expression was observed in the epilepsy group (0.48 ± 0.15). Expression increased following treatment, particularly in the HuMSC group (1.08 ± 0.24), which showed levels comparable to the control group (1.03 ± 0.30). Statistical analysis confirmed that HuMSC treatment significantly increased SOX10 expression compared with the epilepsy, secretome, and exosome groups (p < 0.05). However, no significant difference was observed between the HuMSC and control groups, indicating that HuMSCs restored SOX10 expression to near-normal levels rather than exceeding baseline values.

Similarly, SV2A expression was markedly reduced in the epilepsy group (0.41 ± 0.19) compared with the control group (1.07 ± 0.46). HuMSC treatment (1.12 ± 0.32) restored SV2A expression to levels that were not significantly different from those of the control group. Secretome (0.91 ± 0.45) and exosome (0.63 ± 0.15) treatments produced partial recovery but remained significantly lower than the HuMSC and control groups (p < 0.05).

These findings demonstrate that HuMSC therapy restored both SOX10 and SV2A expression to near-normal control levels, highlighting its superior effect on synaptic and glial-associated gene regulation.

### Effects on cognitive function

Cognitive performance, assessed using the Morris Water Maze test, differed significantly among treatment groups. During the tryout phase, rats in the HuMSC group located the platform most rapidly (4.83 ± 2.13 s), matching the performance of the control group (4.83 ± 2.31 s). In contrast, exosome-treated (18.83 ± 18.18 s) and secretome-treated (21.67 ± 22.17 s) rats required significantly longer times, whereas the epilepsy group showed intermediate performance (9.00 ± 8.40 s).

**Figure 5:**
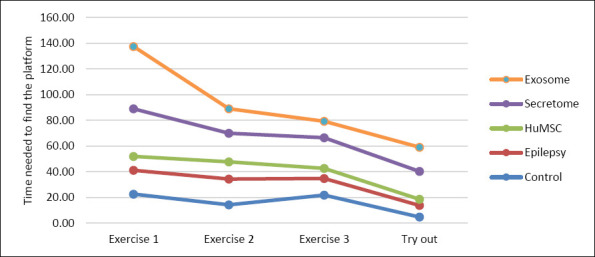
Average time required by rats to locate the platform during training sessions 1, 2, 3, and the tryout phase across different treatment groups. The green line represents the exosome-treated group, the yellow line the secretome-treated group, the gray line the HuMSC-treated group, the orange line the epilepsy-induced group, and the blue line the untreated healthy control group.

Repeated-measures ANOVA demonstrated significant effects of both session (p = 0.007) and treatment (p = 0.005). Post hoc analysis confirmed that the HuMSC group outperformed the other treatment groups throughout all testing sessions (p < 0.01), as shown in Figure 5. A summary of the average time required to locate the platform during each session is presented in Table 1.

**Table 1:** Average time required to locate the platform during training sessions and tryout under different treatments (seconds).

Exercise/Tryout	Control	Epilepsy	HuMSC	Secretome	Exosome
Exercise 1	22.67 ± 28.95	18.50 ± 20.88	10.83 ± 9.91	37.00 ± 26.22	48.50 ± 12.34
Exercise 2	14.33 ± 15.50	20.00 ± 23.08	13.50 ± 11.43	22.16 ± 19.12	19.00 ± 18.05
Exercise 3	21.83 ± 21.89	13.00 ± 17.84	7.83 ± 5.56	23.83 ± 20.12	12.83 ± 15.45
Tryout	4.83 ± 2.31	9.00 ± 8.40	4.83 ± 2.13	21.67 ± 22.17	18.83 ± 18.18
Mean ± SD	15.92 ± 8.29ᵃᵇ	15.13 ± 5.07ᵃᵇ	9.25 ± 3.75ᵃ	26.17 ± 7.28ᵇ	24.79 ± 16.06ᵇ

Different superscript letters indicate statistically significant differences among groups (p < 0.05).

Performance differences among groups shown in Table 1 reflect the distinct impact of each intervention on hippocampal-dependent learning and memory. The HuMSC group consistently exhibited shorter escape latencies across sessions and approached control group performance, reflecting strong neuroprotective and regenerative effects. In contrast, the secretome and exosome groups demonstrated greater variability, with higher mean values and wider standard deviations. This variability may be attributable to weaker or less consistent therapeutic effects, individual differences in response to pilocarpine-induced epilepsy, and the limited regenerative capacity of secretome and exosome therapies compared with intact stem cells. The relatively large standard deviations observed in some groups likely reflect inherent individual variability in behavioral responses to epilepsy induction and treatment, which is common in preclinical models of neurological disorders.

## DISCUSSION

### Neuroprotective effects of HuMSCs on neuronal damage and neuroinflammation

The results of the present study demonstrated that HuMSC therapy significantly reduced neuronal damage, as evidenced by the lower number of damaged cells and lower immunohistochemical scores compared with the epilepsy, secretome, and exosome groups. The HuMSC group showed no significant difference from the control group, confirming its neuroprotective effect. In contrast, the secretome and exosome groups exhibited higher immunohistochemical scores, indicating a comparatively limited therapeutic impact. The superior neuroprotective effect of HuMSCs may be attributed to their ability to differentiate into neural cells, secrete anti-inflammatory mediators such as interleukin-10 and transforming growth factor beta (TGF-β), and promote neurogenesis through the release of trophic factors, including Brain-derived neurotrophic factor and Vascular endothelial growth factor (VEGF) [29, 33, 34]. In addition, HuMSC therapy may inhibit apoptosis and modulate the inflammatory microenvironment more effectively than secretome or exosome treatments.

A critical comparison of these therapeutic approaches highlights important mechanistic differences. Secretome therapy reduces inflammation by modulating cytokine signaling and inhibiting the nuclear factor kappa B pathway, but lacks regenerative capacity, thereby providing only partial protection [33, 35]. Furthermore, soluble proteins, cytokines, and growth factors in the secretome may undergo rapid degradation or clearance, thereby limiting their long-term biological effects. Exosome therapy also faces several limitations, including dose dependency, biodistribution constraints, and potentially inefficient penetration of the blood–brain barrier following intravenous administration. Although exosomes contain bioactive molecules, such as miR-124 and neurotrophic factors, that can modulate inflammation and oxidative stress, they cannot differentiate into neural or glial cells or continuously release trophic factors. These limitations may partly explain the greater neuronal damage observed in the exosome-treated group [36, 37].

The relatively limited therapeutic effect observed in the exosome group contrasts with some previous reports that have demonstrated neuroprotective properties of exosomes. This discrepancy may be associated with differences in dosage, route of administration, treatment timing, or experimental conditions and warrants further investigation. Reactive gliosis, reflected by increased background immunohistochemical staining, was more prominent in the secretome and exosome groups, indicating less effective suppression of neuroinflammation compared with HuMSC therapy [38].

Beyond these observed effects, HuMSCs may exert regenerative actions by modulating signaling pathways such as PI3K/Akt and Wnt/β-catenin, as suggested by previous studies. Although these mechanisms were not directly investigated in the present study, activation of these pathways may promote synaptogenesis, neuronal survival, and glial support, thereby contributing to the superior histological and functional outcomes observed in the HuMSC-treated animals.

### Effects of HuMSCs on SV2A and SOX10 expression

SV2A expression, which is critical for synaptic vesicle function and neurotransmitter release, was significantly reduced in the epilepsy group but was restored by HuMSC treatment to levels comparable to those in the control group. Secretome treatment resulted in only partial recovery, whereas exosome treatment produced intermediate effects. These findings suggest that HuMSCs are more effective in promoting synaptic repair and preserving neuronal connectivity than their cell-free derivatives [39–42].

Similarly, SOX10 expression, an important marker of glial development and myelination, was lowest in the epilepsy group and significantly increased following HuMSC treatment. HuMSC-treated animals exhibited SOX10 expression levels comparable with those of the control group, whereas secretome treatment had little effect and exosome treatment produced only partial improvement. The ability of HuMSCs to modulate inflammation, secrete trophic factors, and potentially differentiate into glial-supporting lineages may explain their superior effect on SOX10 expression [43–45].

Collectively, the restoration of SV2A and SOX10 expression indicates that HuMSC therapy may promote simultaneous recovery of neuronal and glial function. These molecular findings are consistent with the histopathological observations and further support the regenerative potential of HuMSCs in epilepsy.

### Effects of HuMSCs on cognitive function

Cognitive performance, assessed using the Morris Water Maze test, improved most substantially in the HuMSC-treated group, as reflected by significantly shorter platform-finding times compared with the other treatment groups. These findings suggest that HuMSC therapy effectively restores hippocampal-dependent memory and spatial learning, both of which are commonly impaired in epilepsy.

Although the Morris Water Maze is an experimental animal model, its outcomes parallel clinically relevant aspects of cognitive function in humans, including spatial memory, attention, and learning capacity. Therefore, the observed improvements suggest that HuMSC therapy may improve cognitive outcomes in patients with epilepsy while also addressing neuronal injury [39–41].

In contrast, secretome and exosome therapies produced only modest improvements in cognitive performance. While these therapies may provide short-term neuroprotective benefits through paracrine signaling, their effects appear less durable due to limited regenerative capacity and an inability to sustain cellular replacement or support [24, 35, 42].

### Therapeutic implications and mechanistic considerations

The findings of this study support a conceptual distinction between regenerative and paracrine therapeutic mechanisms. Intact HuMSCs appear capable of producing sustained therapeutic effects through both direct cellular interactions and continuous secretion of bioactive molecules. In contrast, secretome and exosome therapies rely predominantly on transient paracrine signaling. This fundamental difference may explain the superior efficacy of HuMSCs across histological, molecular, and behavioral outcomes.

From a translational perspective, the present findings suggest that HuMSC-based therapy may offer clinical value as a neuroprotective and regenerative strategy for epilepsy, particularly in addressing neuronal injury and cognitive impairment beyond seizure suppression alone. Nevertheless, direct extrapolation of the doses used in this rat model to human patients is not currently feasible. Dose optimization, pharmacokinetic studies, biodistribution analyses, and long-term safety evaluations remain necessary before clinical implementation. In addition, regulatory requirements, manufacturing consistency, quality-control standards, and scalability must be carefully considered for both stem cell-based and cell-free biological therapies.

### Study limitations and future perspectives

Although the present study provides valuable comparative insight into regenerative therapies for epilepsy, several limitations should be acknowledged. First, a sample size of 30 rats, distributed across five groups, is acceptable for an exploratory study but may limit statistical power when comparing multiple interventions. Second, cognitive and molecular outcomes were assessed at a single endpoint, which may not fully capture long-term therapeutic effects.

Variability in individual responses to pilocarpine-induced epilepsy and differences in baseline learning abilities may also have contributed to the observed variation in treatment outcomes. Furthermore, the use of a single animal model (*R. norvegicus*) and a single induction method restricts the generalizability of the findings to other forms of epilepsy or neurological disorders.

Another important limitation is that all interventions were administered intravenously. Although this approach facilitates direct comparison among therapies, it may not represent the optimal delivery route for secretome or exosome preparations and could have influenced their biodistribution and therapeutic efficacy within the central nervous system.

In addition, phenotypic validation of HuMSCs and detailed molecular characterization of the secretome and exosomes, including surface-marker profiling, particle-size distribution, and morphological evaluation, were not performed. Consequently, potential differences in the biological properties of these therapeutic products could not be fully verified despite their preparation under standardized laboratory conditions.

Future studies should investigate alternative delivery routes, conduct pharmacokinetic and biodistribution analyses to assess brain uptake, and evaluate long-term therapeutic outcomes. Additional studies should also examine potential interactions between regenerative therapies and conventional antiepileptic drugs to better define their translational and clinical potential.

## CONCLUSION

This study demonstrated that HuMSC therapy provided superior neuroprotective, molecular, and cognitive benefits compared with secretome and exosome treatments in a pilocarpine-induced epilepsy model. Histopathological evaluation showed that HuMSC-treated animals exhibited substantially lower neuronal damage than untreated epilepsy, secretome-treated, and exosome-treated groups, with values approaching those of healthy controls. At the molecular level, HuMSC therapy restored SV2A and SOX10 expression to near-normal levels, indicating improved synaptic integrity, neuronal communication, and glial support. Furthermore, HuMSC-treated animals exhibited significantly better cognitive performance in the Morris Water Maze test, as reflected by shorter escape latency and improved spatial learning ability.

The findings suggest that HuMSCs provide therapeutic advantages through a combination of regenerative and paracrine mechanisms, whereas secretome and exosome therapies appear to rely predominantly on transient paracrine effects. This distinction may explain the superior efficacy of HuMSCs in reducing neuronal injury, enhancing molecular recovery, and improving cognitive outcomes. From a practical perspective, these results support the potential application of HuMSC-based therapy as a regenerative strategy for epilepsy that may address both neuronal damage and cognitive dysfunction, extending beyond conventional approaches that primarily focus on seizure control.

A major strength of this study is the direct comparison of cell-based and cell-free regenerative therapies within the same experimental epilepsy model using integrated histopathological, molecular, and behavioral assessments. This comprehensive approach enabled a more robust evaluation of therapeutic efficacy than studies examining these interventions independently.

However, several limitations should be acknowledged. The relatively small sample size, use of a single animal model and epilepsy induction method, single-endpoint assessment of outcomes, and lack of detailed phenotypic characterization of HuMSCs, secretome, and exosomes may limit the generalizability of the findings. In addition, pharmacokinetics, biodistribution, and long-term therapeutic effects were not evaluated. The exclusive use of intravenous administration may also have influenced the efficacy of secretome and exosome delivery to the central nervous system.

Future studies should investigate optimized dosing regimens, alternative delivery routes, long-term therapeutic outcomes, and the pharmacokinetic behavior of regenerative therapies in epilepsy. Detailed characterization of therapeutic products and evaluation of their interactions with conventional antiepileptic drugs are also warranted. Furthermore, validation in larger animal models and clinical studies will be essential to determine safety, efficacy, and translational feasibility.

Overall, the present findings indicate that HuMSCs possess greater therapeutic potential than secretome and exosome preparations for the treatment of epilepsy. By simultaneously reducing neuronal damage, restoring molecular markers associated with synaptic and glial function, and improving cognitive performance, HuMSCs represent a promising regenerative therapeutic approach that merits further investigation for future clinical application in epilepsy management.

## DATA AVAILABILITY

The supplementary data can be made available from the corresponding author upon request.

## AUTHORS’ CONTRIBUTIONS

SH: Conceptualization, investigation, formal analysis, figure preparation, data interpretation, and writing – original draft. IB: Conceptualization, data interpretation, literature review, and writing – original draft. YR: Data curation, validation, formal analysis, data interpretation, and supervision. II: Methodology, resources, investigation, data interpretation, and supervision. RI: Project administration, supervision, data interpretation, and critical review of the manuscript. All authors have read, reviewed, and approved the final version of the manuscript.

## COMPETING INTERESTS

The authors declare that they have no competing interests.

## PUBLISHER’S NOTE

Veterinary World remains neutral with regard to jurisdictional claims in the published institutional affiliations.
